# Variation among arthropod taxa in the amino acid content of exoskeleton and digestible tissue

**DOI:** 10.1002/ece3.10348

**Published:** 2023-07-24

**Authors:** Jamie T. Reeves, Colton Herzog, Cody L. Barnes, Craig A. Davis, Samuel D. Fuhlendorf, Shawn M. Wilder

**Affiliations:** ^1^ Department of Integrative Biology Oklahoma State University Stillwater Oklahoma USA; ^2^ Department of Biology Utah State University Logan Utah USA; ^3^ Department of Natural Resource Ecology and Management Oklahoma State University Stillwater Oklahoma USA

**Keywords:** amino acid, arthropod, digestible tissue, exoskeleton

## Abstract

Arthropod consumption provides amino acids to invertebrates and vertebrates alike, but not all amino acids in arthropods may be digestible as some are bound in the exoskeleton. Consumers may not be able to digest exoskeleton in significant amounts or avoid it entirely (e.g., extraoral digestion). Hence, measures that do not separate digestible amino acids from those in exoskeleton may not accurately represent the amino acids available to consumers. Additionally, arthropods are taxonomically diverse, and it remains unclear if taxonomic differences also reflect differences in amino acid availability. Thus, we tested: (1) if there were consistent differences in the content and balance of amino acids between the digestible tissue and exoskeleton of arthropods and (2) if arthropod Orders differ in amino acid content and balance. We measured the amino acid content (mg/100 mg dry mass) and balance (mg/100 mg protein) of whole bodies and exoskeleton of a variety of arthropods using acid hydrolysis. Overall, there was higher amino acid content in digestible tissue. There were also significant differences in the amino acid balance of proteins in digestible tissue and exoskeleton. Amino acid content and balance also varied among Orders; digestible tissues of Hemiptera contained more of some essential amino acids than other Orders. These results demonstrate that arthropod taxa vary in amino acid content, which could have implications for prey choice by insectivores. In addition, exoskeleton and digestible tissue content differ in arthropods, which means that whole body amino acid content of an arthropod is not necessarily a predictor of amino acid intake of a predator that feeds on that arthropod.

## Graphical abstract

Exoskeleton is a ubiquitous component of arthropods, which many consumers cannot digest. Arthropod exoskeleton differs from digestible tissue in amino acid balance and content. Amino acid balance and content also vary considerably among arthropod taxa.

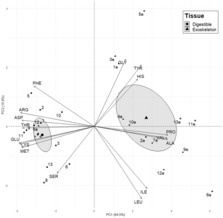

## 1 INTRODUCTION

Animals require specific balances of macronutrients (fat, carbohydrates, proteins), dietary essential nutrients (amino acids, fatty acids), micronutrients (vitamins, trace elements), and other substances (digestive aids such as grit, etc.) to maximize fitness (Simpson & Raubenheimer, 2012; Sterner & Elser, 2002). Most work on nutritional ecology has focused on bulk macronutrients (e.g., carbohydrate, lipid, protein) and macroelements (e.g., CHON, P, K). Yet, recent advances suggest micronutrients also affect life history traits of organisms or ecological processes (Anderson et al., 2004; Carnicer et al., 2015; Clay et al., 2017; Filipiak & Filipiak, 2022; Kaspari, 2020, 2021; Kaspari et al., 2014, 2016, 2021; Paseka et al., 2019; Prather et al., 2020; Welti et al., 2019). Animals often require specific dietary essential nutrients because they lack the ability to synthesize them de novo, and consuming adequate amounts of dietary essential nutrients positively impacts consumer performance. For example, fatty acid content of food can have significant impacts on consumer growth, survival, and reproduction (Twining et al., 2018, 2021), and amino acid content of food items can also influence consumer reproduction (Grandison et al., 2009), survival (Arganda et al., 2017; Wilder & Schnieder, 2017), and prey choice (Greenstone, 1979).

Amino acids are the molecular subunits of proteins, but also exist in their free states (or bound to transporters) to meet the demands of protein synthesis and for other metabolic products (e.g., S‐Adenosyl methionine). Of the 20 amino acids encoded in DNA, the carbon skeleton of nine essential amino acids (EAA) cannot be synthesized in all animal cells (Abderhalden, 1912; Rose, 1957; Wu, 2013). The nonessential amino acids (NEAA) are all thought to be synthesized by animals, though some are classified as conditionally essential (CEAA) because their synthesis requires EAAs as precursors (Aggrey et al., 2017; Stinapuk, 2020; Wu, 2014; Wu et al., 2014). Alternatively, the rate of NEAA synthesis may not account entirely for metabolic demand (Robbins, 1993; Scott et al., 1982; Murphy, 1996; Klasing, 1998). For example, evidence suggests that some animals benefit in growth or reproduction from dietary supplementation of NEAAs that play important roles in biochemical pathways, rather than depending on synthesis alone (Hou et al., 2015, 2016; Hou & Wu, 2017; Peoples et al., 1994; Wilder, 2011; Wu, 2010, 2013, 2014; Wu et al., 2014). Thus, definitions of EAA, CEAA, and NEAA may vary and depend on species, life‐stage, sex, reproductive status, feeding guild, or internal symbiotic interactions (Douglas, 1998; Finkelstein & Martin, 1984; Klasing, 1998; Langlois & McWilliams, 2022; Nygaard et al., 2011; Sterkel et al., 2016; Suen et al., 2011; Tapiero et al., 2002; Wu et al., 2014). Regardless, the amount and balance of amino acids can have important consequences for animal fitness (Ramsay & Houston, 1998).

Arthropoda is the largest, most diverse phylum of animals, and arthropods are consumed in large quantities by invertebrates and vertebrates (Bell, 1990; Nyffeler & Birkhofer, 2017). Variation in the amino acid content of arthropods may play a role in the performance of consumers, but most studies of arthropod amino acid content are limited to commercially available species (Boulos et al., 2020; Ritvanen et al., 2020; Smets et al., 2021). There are few data available to examine even basic questions, such as whether there are consistent differences in amino acid content between arthropod taxa (Ramsay & Houston, 2003). Knowledge of the availability of arthropod nutrients is critically important amid variation in the abundance and diversity of arthropods in space and time (Crossley et al., 2020; Fenoglio et al., 2020; Lister & Garcia, 2018; Salcido et al., 2020; Sánchez‐Bayo & Wyckhuys, 2019, 2021; Tallamy & Shriver, 2021, but see also Thomas et al., 2019; Willig et al., 2019). For example, Crossley et al. (2020) observed no net change in abundance or diversity of arthropods across North America from long‐term research sites (4–36 years) but report local or regional trends that may influence food availability for consumers at variable spatial scales. To understand the consequences for consumers, the nutritional costs and gains of arthropod consumption for individual consumers, and the resulting flow of elements and molecules through ecosystems, must be more thoroughly understood.

Exoskeleton is a defining feature of arthropods that varies in amount within and among taxonomic groups (Andersen et al., 1995; Kramer et al., 1995; Lease & Wolf, 2010; Reeves et al., 2021). In insects and spiders, exoskeleton consists of a mixture of chitin and protein, the latter of which is locked in the matrix of the exoskeleton (Andersen, 2010). Additionally, several amino acids may be particularly important for sclerotization (secondary hardening) of the exoskeleton. For instance, quinones destined for oxidative incorporation into the chitin matrix are ultimately derived from tyrosine, and quinone products depend on the availability of nucleophilic side chains (e.g., histidine, lysine, arginine) for localization within the chitin matrix and crosslink formation (Andersen, 2008, 2010; Andersen & Roepstorff, 2007; Andersen et al., 1995; Sugumaran, 1998). However, it remains unclear how variation in exoskeleton proteins contributes to the relative amount and balance of amino acids in exoskeleton compared to digestible tissue and across arthropod taxa from a consumer's perspective.

Overall, nutritional gains from terrestrial arthropod exoskeleton may be minimal. Chitin digestibility varies widely among consumers but is relatively low in most species that have been examined (Akaki & Duke, 1999; Bell, 1990; Hossain & Blair, 2007; Jackson et al., 1992; Kay & Sheine, 1979; Weiser et al., 1997). For example, some seabirds can digest chitin (Jackson et al., 1992) but most other species appear limited (Akaki & Duke, 1999; Cohen, 1995; Weiser et al., 1997). Grinding may slightly increase nutritional gains from exoskeleton but contribution to the total diet is likely minimal (Hossain & Blair, 2007; Kay & Sheine, 1979). Although cecal fermentation of plant carbohydrates and the assistance of microbial action has been studied in some birds (Clench & Mathias, 1995), the chitin digestion capacity and potential for nutritional gains from microbial fermentation have not been measured. Lack of chitin digestion is especially apparent in predatory arthropods that feed using extraoral digestion, which involves extracting digestible tissue from prey and leaving behind unconsumed piles of exoskeleton (i.e., spiders, scorpions, predatory hemipterans, etc.) (Barnes et al., 2019; Cohen, 1995, 1998; Foelix, 1996; Walter et al., 2017). Hence, it is important to separate exoskeleton from digestible tissue when studying the nutritional ecology of arthropodivores (Bell, 1990; Jonas‐Levi & Martinez, 2017).

We analyzed the amino acid content and balance of the whole bodies and exoskeletons of a variety of arthropods to address two objectives. The first objective of this research examined differences in amino acid content and balance between exoskeleton and digestible tissue of arthropods to better understand the nutritional ecology of arthropodivores. The second objective compared amino acid content and balance among three arthropod Orders to better understand variation in amino acid availability across groups of potential prey taxa.

## 2 METHODS

### 2.1 Exoskeleton versus digestible tissue

The methods for this part of the study were the same as Wilder and Barnes (2023). Wilder and Barnes (2023) used the data on total protein content of these arthropods calculated from the amino acid analysis but did not analyze individual amino acids. In this study, we analyzed the amino acids data. We used adults of 13 species of arthropods, each from a different Order, in this portion of the study. We included one arachnid (Araneae, Araneidae, *Neoscona crucifera*, *n* = 5), one crustacean (Isopoda, Armadilidiidae, *n* = 7), and 11 different orders of insects (Blattodea, Ectobiidae, *Blattella germanica*, *n* = 7; Coleoptera, Scarabeidae, *Cotinus nitida*, *n* = 2; Diptera, Tabanidae, *n* = 4; Ephemeroptera, Ephemeridae, *Hexagenia* sp., *n* = 5; Hemiptera, Coreidae, *Anasa tristis*, *n* = 6; Hymenoptera, Vespidae, *n* = 7; Lepidoptera, Nymphalidae, *Asterocampa celtis*, *n* = 4; Mantodea, Mantidae, *Stagmomantis sp.*, *n* = 2; Odonata, Libellulidae, *Plathemis sp.*, *n* = 2; Orthoptera, Acrididae, *Syrbula admiralis*, *n* = 2; Phasmatodea, Diapheromeridae, *n* = 2). Sample sizes represent the number of individuals combined to measure the amino acid content of whole arthropods and the number of individuals combined to measure the amino acid content of exoskeleton. Species were used as the unit of replication in analyses and, hence, there were 13 replicates for tests of these hypotheses. Species in this section were chosen to represent a diversity of arthropod Orders and adult body forms, and because of their commercial availability.

We first dried and removed lipids from all samples using the gravimetric method with chloroform (Cuff et al., 2021). We then used one or more individuals of each species for determination of exoskeleton content. Briefly, we dried arthropod samples (i.e., 60°C for 24 h), weighed them, gently broke open the exoskeleton to allow NaOH to enter the body, soaked samples in 0.1 M NaOH, washed them with water, redried them, and weighed the remaining biomass, which is exoskeleton (Cuff et al., 2021). We used a mixer mill with stainless steel beads to grind the exoskeleton and also ground separate samples of whole arthropods of each species into a powder to prepare them for acid hydrolysis.

Amino acid analysis measured the content of both the whole body and the exoskeleton of each of the 13 arthropod species. AAA Service Lab conducted amino acid analysis using a Hitachi L8900 Amino Acid Analyzer. This analysis quantified concentrations of the 16 most common amino acids, but not cysteine or tryptophan. In arthropods, these two amino acids are typically the least abundant and comprise a very small percentage of total amino acid content (Boulos et al., 2020; Ritvanen et al., 2020; Smets et al., 2021). Hence, we assumed that the results were a close approximation of the amino acid content of the samples. Measurements of amino acids in the exoskeleton and the whole body were taken on separate pooled samples for each species.

### 2.2 Differences among arthropod orders

To address the similarity in amino acid content and balance between arthropod Orders, we collected all samples from a single study site to represent different species that might be available for a single predator. The study site, Packsaddle Wildlife Management Area, is located in Ellis County, Oklahoma on the western edge of the Central Great Plains Level III Ecoregion (U.S. Environmental Protection Agency, 2013; Wiken et al., 2011), and experiences considerable spatial and temporal variation in disturbance conditions, vegetation composition, temperature, precipitation, and soil texture and nutrient conditions (Arndt, 2003; Carter & Gregory, 1996; Oklahoma Climatological Survey and Mesonet, 2023; Peterson & Boyd, 1998; Rakowski et al., 2019; Reeves et al., 2022). We also only identified arthropods to morphospecies, as this is likely the level of identification that visually hunting predators might use. The methods for arthropod collection, handling, and storage in this portion of the study were the same as Reeves et al. (2021).

Like Wilder and Barnes (2023), Reeves et al. (2021) used the amino acid data to calculate and analyze total protein content, but did not analyze amino acid data in their study. The methods for sample preparation and chemical analysis were identical to those in the previous section. This dataset included 20 morphospecies from three insect Orders: Coleoptera (*n* = 6), Hemiptera (*n* = 7), and Orthoptera (*n* = 7). We chose species to use as representatives of each Order first by whether there was enough biomass available for chemical analysis, and then to maximize variation in body form (and potentially amino acid balance) within each taxonomic group. We analyzed amino acid content of all 20 whole arthropods and one combined sample of exoskeleton for each order. A problem occurred during amino acid analysis in the combined exoskeleton samples of the comparison among orders that resulted in no data for isoleucine and leucine in the combined exoskeleton samples, which prevented us from calculating digestible isoleucine and leucine.

Using this dataset, we tested if there were differences among Orders in amino acid content and balance using morphospecies as replicates. Amino acid analysis uses acid hydrolysis to digest samples, which solubilizes amino acids both in soft tissue and those bound in the chitinous matrix of the exoskeleton. However, most consumers cannot digest exoskeleton and the amino acids contained therein. Hence, we calculated digestible (i.e., soft tissue) amino acid content as a measure of amino acids comparable to that consumed by organisms which cannot digest exoskeleton (e.g., spiders, terrestrial birds, mammals, etc.). We calculated digestible amino acid content of samples as the difference between the amino acid content of the whole arthropod and the amino acids in the exoskeleton (i.e., digestible AA = whole arthropod AA—exoskeleton AA * proportion exoskeleton).

### 2.3 Data and analysis

For this work, we separately defined amino acid content and balance. Content was defined as the concentration of each amino acid per 100 mg dry mass of the whole organism or tissue (i.e., either digestible tissue or exoskeleton). Content is a measure of the amount of each amino acid in a whole organism or tissue, controlling for a standard mass of that tissue. Balance was defined as the concentration of each amino acid per 100 mg of protein in that animal or tissue to compare proteins themselves more directly. Hence, balance only considers the proteins in an organism and measures the amount of each amino acid in that protein (Ramsay & Houston, 1998).

Datasets for comparisons of digestible tissue versus exoskeleton, and among arthropod orders are presented here in content (mg AA/100 mg dry mass) and balance (mg AA/100 mg protein). For amino acids quantified in this work, due to the limited knowledge regarding amino acid synthesis across arthropod taxa, we defined EAA, CEAA, and NEAA as follows: EAA include histidine, isoleucine, leucine, lysine, methionine, phenylalanine, threonine, tyrosine, and valine, CEAA include arginine, glutamic acid, glycine, and proline, and NEAA include alanine, aspartic acid, and serine (Douglas, 1998; Hou et al., 2015, 2016; Hou & Wu, 2017; Nygaard et al., 2011; Sterkel et al., 2016; Stinapuk, 2020; Suen et al., 2011; Tapiero et al., 2002; Wu, 2013, 2014; Wu et al., 2014). Untransformed datasets failed to meet assumptions of normality, and log and square root transformation did not result in normal distribution. Thus, we used untransformed data for analysis.

We used Levene's test to examine the datasets for differences in variance of individual amino acid content and balance between digestible tissue and exoskeleton as well as among Orders. For amino acids with significantly differing variance between groups (i.e., digestible vs. exoskeleton or among Orders), we subsequently used Welch's ANOVA to test if average content and balance differed between groups for both datasets. We then used the Games‐Howell post‐hoc test to examine inter‐group differences. For amino acids without significant differences in variance between groups, we used ANOVA and Tukey HSD where appropriate.

We used a principal component analysis (PCA) to visualize and reduce dimensionality of datasets to test overall differences in amino acid content and balance between digestible tissue and exoskeleton as well as among Orders. We extracted the PC axes explaining the most variation and used them to test for an effect of grouping (i.e., tissue or taxa). We used ANOVA to test extracted PC scores for differences across groups. We subsequently created linear models using PC scores to test for overall relationships between amino acid profile and grouping (i.e., tissue or taxa). Additionally, to validate PCA results, we conducted an analysis of multivariate homogeneity of group dispersions (variances) and a permutational multivariate analysis of variance (PERMANOVA) using Bray–Curtis dissimilarity matrices to test if grouping (i.e., tissue or taxa) resulted in differences in spread or location in sampling space (Anderson, 2005, 2017; Oksanen et al., 2022). We used R statistical software ver. 4.0.0 to analyze all data (R Core Team, 2020) and used package “vegan” to perform multivariate tests (Oksanen et al., 2022).

## 3 RESULTS

### 3.1 Exoskeleton versus digestible tissue

**FIGURE 1:**
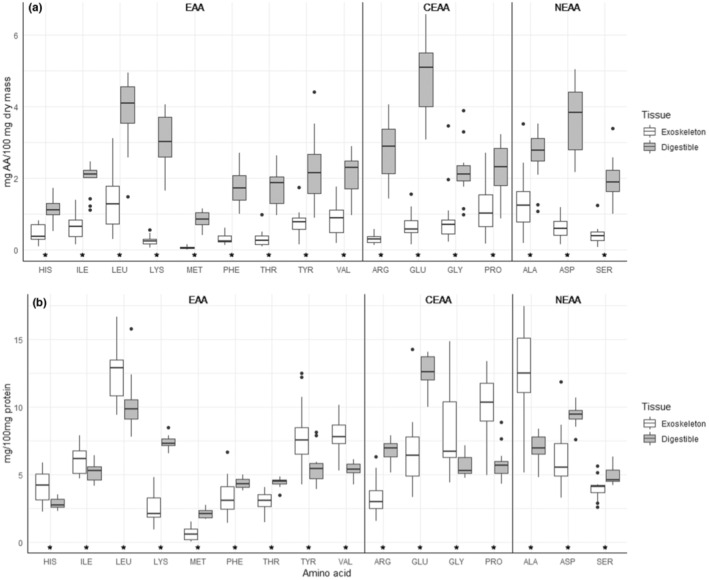
Content 16 individual amino acid residues measured in exoskeleton and metabolizable tissue of 13 orders of arthropod (*n* = 13). Data are presented as (a) amino acid content (mg/100 mg dry mass) and (b) balance (mg/100 mg Protein). (*) Indicates a significant difference between tissues for that amino acid.

The amino acid content (i.e., mg/100 mg dry mass) of exoskeleton contained less of all 16 amino acids than digestible tissue (Table 1; Figure 1a). However, when controlling for the protein content of tissues, there were differences in the balance (i.e., mg/100 mg protein) of amino acids (Table 1; Figure 1b). Levene's test detected differences in variance in nine amino acids between exoskeleton and digestible tissue when analyzed as content (i.e., mg/100 mg dry mass) (Table S1A) and 10 amino acids when analyzed as balance (i.e., mg/100 mg protein) (Table S1B). When analyzing total amino acid content of samples, digestible amino acid content was more variable than exoskeleton (Table S1A; Figure 1a), whereas after controlling for total protein (balance), exoskeleton amino acids were more variable than digestible amino acids (Table S1B; Figure 1b).

**TABLE 1:** Test statistics for ANOVAs performed on individual amino acids to test differences between soft tissue and exoskeleton amino acid content.

Amino acid	(A) Protein proportion					(B) Mass proportion				
	df	rdf	MSE	*F*	*p*	df	rdf	MSE	*F*	*p*
Ala	1	14.2	w	31.3	<.001	1	24	0.7	14.8	<.001
Arg	1	24	1.3	58.6	<.001	1	12.7	w	105.05	<.001
Asp	1	14.4	w	19.9	<.001	1	14.6	w	121	<.001
Glu	1	24	4.9	44.1	<.001	1	14.9	w	156.3	<.001
Gly	1	13.3	w	7.8	<.001	1	24	0.7	15.0	<.001
His	1	14.3	w	12.8	<.001	1	24	0.09	31.8	<.001
Ile	1	24	0.7	8.5	<.001	1	24	0.2	74.1	<.001
Leu	1	24	4.7	6.0	<.001	1	24	0.8	51.1	<.001
Lys	1	16.7	w	206.3	<.001	1	12.8	w	150.5	<.001
Met	1	24	0.2	89.8	<.001	1	12.9	w	143.1	<.001
Phe	1	13.7	w	6.0	<.001	1	14.3	w	96.6	<.001
Pro	1	17.9	w	31.2	<.001	1	24	0.5	15.2	<.001
Ser	1	24	0.6	10.6	<.001	1	16.5	w	58.5	<.001
Thr	1	17.3	w	38.9	<.001	1	17.2	w	87.6	<.001
Tyr	1	17.2	w	9.1	<.001	1	15.6	w	24.5	<.001
Val	1	15.7	w	39.2	<.001	1	24	0.3	33.6	<.001

*Note*: Amino acid data were calculated as (A) a proportion of total protein (mg/100 mg protein) and (B) as a proportion of dry mass (mg/100 mg dry mass). (w) in the MSE column indicates Welch's ANOVA and hence no MSE value.

**FIGURE 2:**
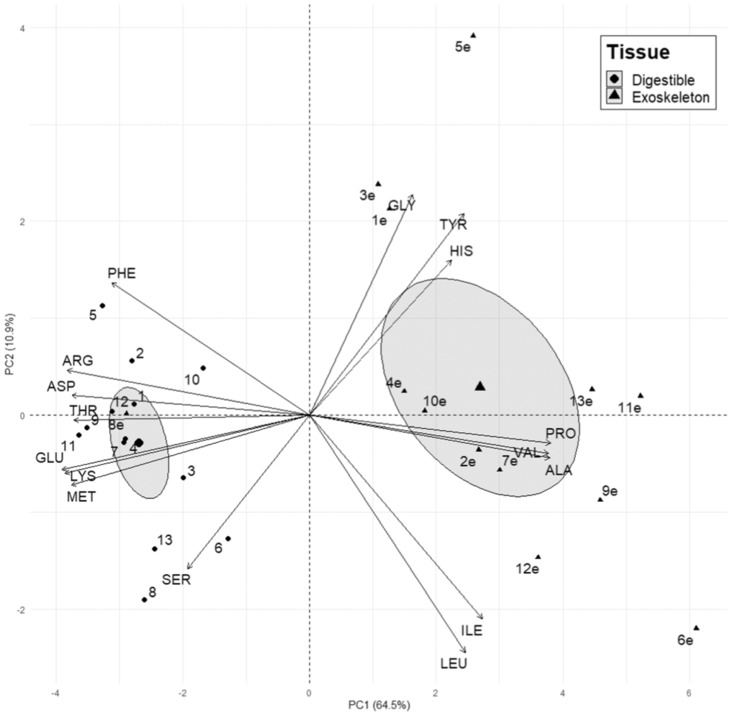
Principal component analysis of metabolizable and exoskeleton amino acid balance of 16 residues as a proportion of protein (mg/100 mg Protein). Large points indicate group centroids, and ellipses indicate 95% confidence intervals. Point label indicates corresponding arthropod Order: (1) Araneae, (2) Blattodea, (3) Coleoptera, (4) Diptera, (5) Ephemeroptera, (6) Hemiptera, (7) Hymenoptera, (8) Isopoda, (9) Lepidoptera, (10) Mantodea, (11) Odonata, (12) Orthoptera, and (13) Phasmatodea. Inclusion of “e” in point label indicates exoskeleton. A significant effect of tissue was observed on PC1 (ANOVA; *F* = 65.2, *p* < .0001).

The PCA of amino acid balance (i.e., mg/100 mg protein) revealed differences in the overall amino acid profile of arthropod tissues (Figure 2). PC1 scores, which explained 64% of observed variation, differed significantly between exoskeleton and digestible tissue (Figure 2). We also conducted linear regression of digestible and exoskeleton PC1 scores to test if there were relationships between the amino acid balance of these tissues but did not detect a significant relationship (Figure S1A). The analysis of multivariate homogeneity of group dispersions (variance) performed on amino acid balance (i.e., mg/100 mg protein) indicated that dispersion differed between exoskeleton and digestible tissue (*betadisper—anova*; *F* = 14.8, *p* = .0008). However, due to the distinct separation of exoskeleton and digestible tissue 95% confidence intervals in PC space (Figure 2), and because of the relative insensitivity of *adonis* to differences in dispersion (Oksanen et al., 2022), PERMANOVA was still performed. PERMANOVA results indicated that digestible tissue and exoskeleton centroids differed significantly (permutations = 999, df = 1, *R* ^2^ = .6, *F* = 31.2, *p* = .001).

The PCA of amino acid content (i.e., mg/100 mg dry mass) also revealed differences in the overall amino acid profile of arthropod tissues (Figure S2). PC1 scores, which explained 89% of observed variation, differed significantly between exoskeleton and digestible tissue (Figure S2). We also conducted linear regression of digestible and exoskeleton PC1 scores to test if there were relationships between the amino acid balance of these tissues but did not detect a significant relationship (Figure S1B). The analysis of multivariate homogeneity of group dispersions (variance) performed on amino acid content (i.e., mg/100 mg dry mass) indicated that dispersion differed between exoskeleton and digestible tissue (*betadisper—anova*; *F* = 8.9, *p* = .005). PERMANOVA results indicated that digestible tissue and exoskeleton centroids differed significantly (permutations = 999, df = 1, *R* ^2^ = .6, *F* = 39.0, *p* = .001).

### 3.2 Taxonomic variation

**FIGURE 3:**
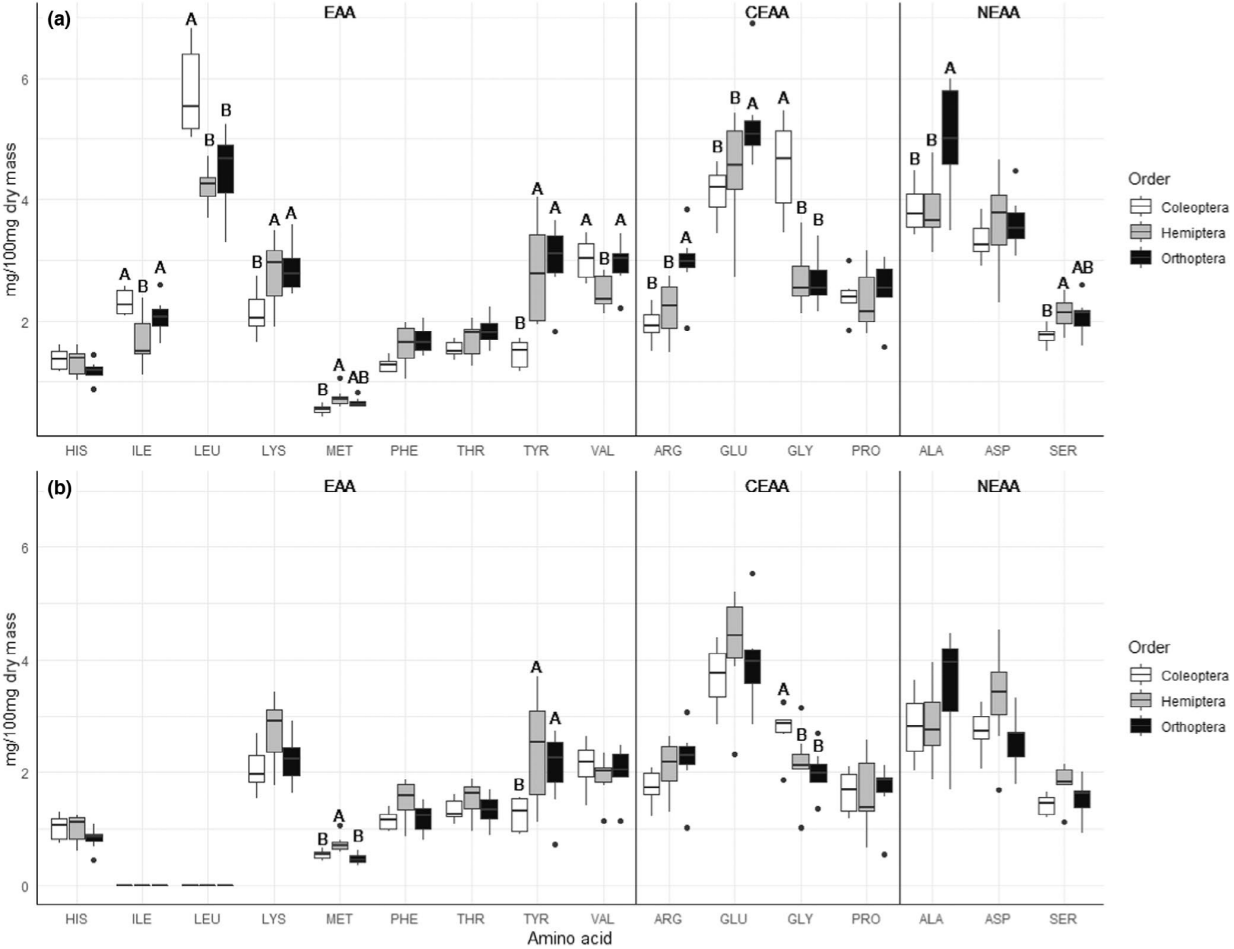
Content of individual amino acid residues measured in three Orders of arthropod: Coleoptera (*n* = 6), Hemiptera (*n* = 7), and Orthoptera (*n* = 7). Amino acid data were calculated as a proportion of dry mass (mg/100 mg dry mass) from (a) whole arthropods and (b) digestible tissue. Groups within an individual amino acid not connected by the same letter differ significantly, but letters are not comparable across amino acids. Unlabeled amino acids were not affected by Order.

In comparisons of individual amino acids, the average content (i.e., mg/100 mg dry mass) of 11 amino acids in whole arthropods differed among the three insect Orders, and three amino acids in digestible tissue differed among orders (Table 2; Figure 3). The average balance (i.e., mg/100 mg protein) of 14 amino acids in whole arthropods differed among the three insect Orders, and 10 amino acids in digestible tissue differed among orders (Table 3; Figure S3). We observed differences in variance in the EAA tyrosine and the CEAA glycine in whole arthropods and tyrosine in digestible tissues when analyzing amino acid content (i.e., mg/100 mg dry mass; Table S2). When analyzed as amino acid balance (i.e., mg/100 mg protein), we observed differences in variance in the EAAs histidine, isoleucine, and tyrosine and the CEAA glycine in whole arthropods and histidine in digestible tissues (Table S3).

**TABLE 2:** Test statistics for ANOVAs performed on individual amino acids to test differences between arthropod orders in residue content.

Amino acid	(A) Whole body					(B) Digestible				
	df	rdf	MSE	*F*	*p*	df	rdf	MSE	*F*	*p*
Ala	2	17	0.5	6.9	.006	2	17	0.6	1.7	.2
Arg	2	17	0.2	8.1	.003	2	17	0.3	1.6	.2
Asp	2	17	0.3	0.6	.6	2	17	0.4	2.6	.1
Glu	2	17	0.6	4.1	.04	2	17	0.7	0.8	.5
Gly	2	10.1	w	10.1	.002	2	17	0.3	3.6	.05
His	2	17	0.04	1.8	0.2	2	17	0.05	1.5	.2
Ile	2	17	0.1	5.6	.01	—	—	—	—	—
Leu	2	17	0.4	11.8	<.001	——	—	—	—	—
Lys	2	17	0.2	4.6	.03	2	17	0.2	3.2	.1
Met	2	17	0.01	4.5	.03	2	17	0.01	8.5	.003
Phe	2	17	0.08	3.2	.1	2	17	0.08	3.2	.1
Pro	2	17	0.2	0.2	.8	2	17	0.3	0.002	1
Ser	2	17	0.07	3.7	.05	2	17	0.1	2.9	.1
Thr	2	17	0.05	2.7	.1	2	17	0.08	1.2	.3
Tyr	2	9.7	w	22.9	<.001	2	10.0	w	6.6	.01
Val	2	17	0.1	4.8	.02	2	17	0.2	0.4	.6

*Note*: Amino acid data were calculated as a proportion of dry mass (mg/100 mg dry mass) from (A) whole arthropods and (B) digestible tissue. *p*‐values <.05 are considered significant. (w) in the MSE column indicates use of Welch's ANOVA due to unequal variance.

**TABLE 3:** Test statistics for ANOVAs performed on individual residues to test differences between arthropod orders in amino acid balance.

Amino acid	(A) Whole body					(B) Digestible				
	df	rdf	MSE	*F*	*p*	df	rdf	MSE	*F*	*p*
Ala	2	17	1.5	5.0	.02	2	17	2.0	4.4	.03
Arg	2	17	0.4	13.8	<.001	2	17	0.6	5.5	.01
Asp	2	17	1.1	1.3	.3	2	17	1.3	3.4	.1
Glu	2	17	1.3	4.1	.03	2	17	1.8	1.3	.3
Gly	2	9.1	w	25.3	<.001	2	17	0.6	22.2	<0.001
His	2	7.9	w	10.6	.006	2	8.8	w	6.0	.02
Ile	2	8.7	w	11.0	.004	—	—	—	—	—
Leu	2	17	1.9	15.3	<.001	—	—	—	—	—
Lys	2	17	0.5	9.3	.002	2	17	0.5	7.1	.006
Met	2	17	0.05	7.8	.004	2	17	0.07	9.1	.002
Phe	2	17	0.1	9.4	.002	2	17	0.2	7.4	.005
Pro	2	17	0.7	0.1	.9	2	17	1.0	0.009	1
Ser	2	17	0.1	15.7	<.001	2	17	0.2	10.8	<.001
Thr	2	17	0.09	3.7	.05	2	17	0.1	2.7	.1
Tyr	2	9.7	w	27.3	<.001	2	17	2.0	7.9	.004
Val	2	17	0.3	8.5	.003	2	17	0.3	3.5	.05

*Note*: Amino acid data were calculated as a proportion of total protein (mg/100 mg protein) from (A) whole arthropods and (B) digestible tissue. *p*‐values <.05 are considered significant. (w) in the MSE column indicates use of Welch's ANOVA due to unequal variance.

**FIGURE 4:**
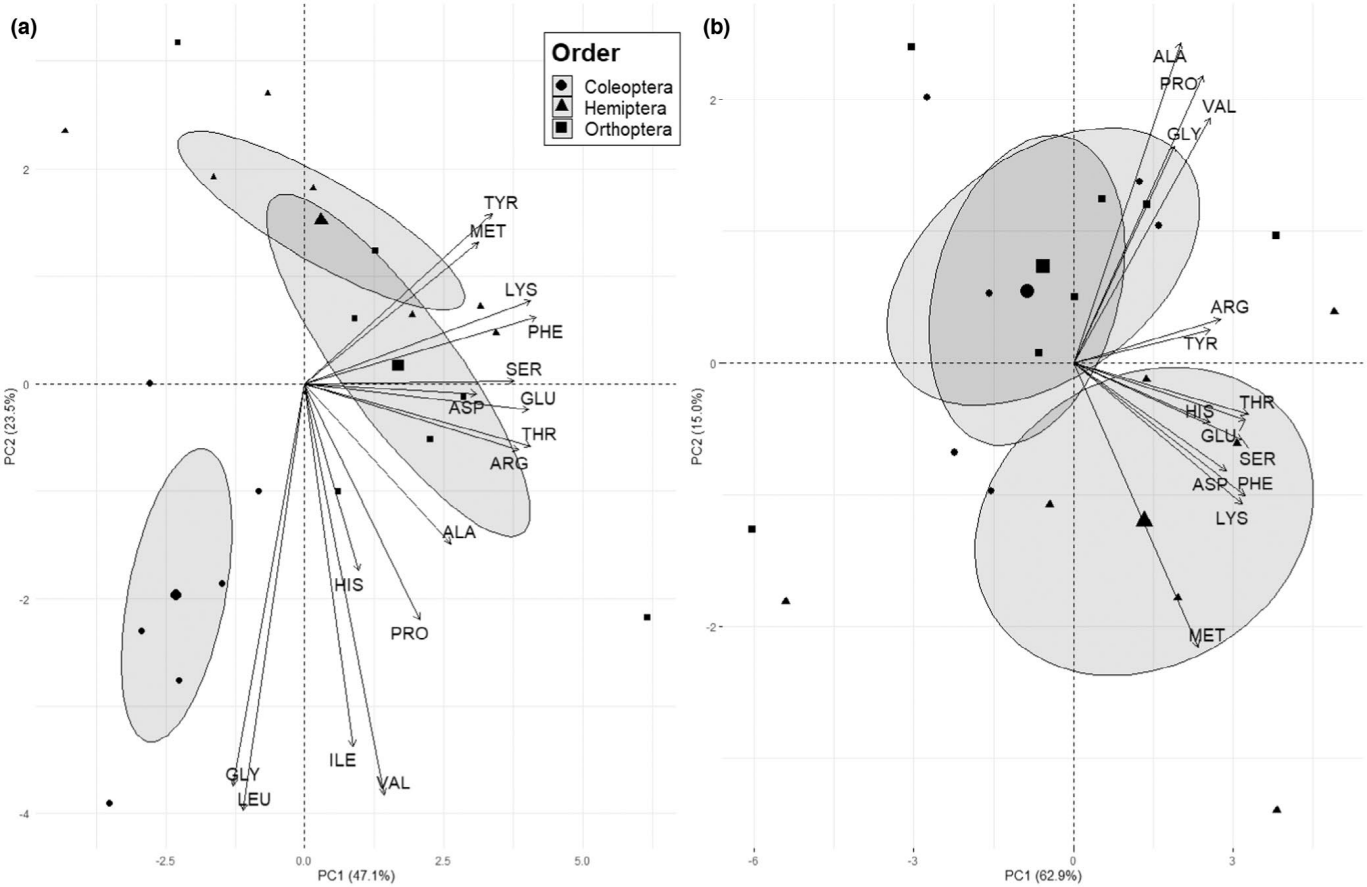
Principal component analysis of whole arthropod amino acid content of 16 amino acids as a proportion of dry mass (mg/100 mg dry mass) for three arthropod orders from (a) whole arthropods and (b) digestible tissue. For (a) whole arthropods, Order significantly affected PC1 (ANOVA; *F* = 22.8, *p* = 1.6 × 10^−5^) and PC2 scores (ANOVA; *F* = 3.6, *p* = .05). For (b) digestible tissue, Order significantly affected PC2 scores (ANOVA; *F* = 5.5, *p* = .01) but not PC1 scores (*p* = .4).

The PCA of whole amino acid content (i.e., as a proportion of dry mass) revealed differences in overall content between arthropod orders (Figure 4a). We observed significant effects of Order on PC1 and PC2 scores for whole arthropods. Analysis of multivariate homogeneity of dispersions did not detect differences in dispersion between Orders (*betadisper—permutest*; permutations = 999, df = 2, *F* = 0.9, *p* = .5). PERMANOVA rejected the null hypothesis, indicating that Order centroids are not equivalent (permutations = 999, df = 2, *R* ^2^ = .4, *F* = 5.7, *p* = .001). For digestible tissue, Order significantly affected only PC2 score (Figure 4b). However, we did not observe differences in multivariate dispersion (*betadisper—permutest*; permutations = 999, df = 2, *F* = 0.2, *p* = .9), and PERMANOVA failed to reject the null hypothesis that centroids are equivalent (permutations = 999, df = 2, *F* = 1.4, *p* = .3).

**FIGURE 5:**
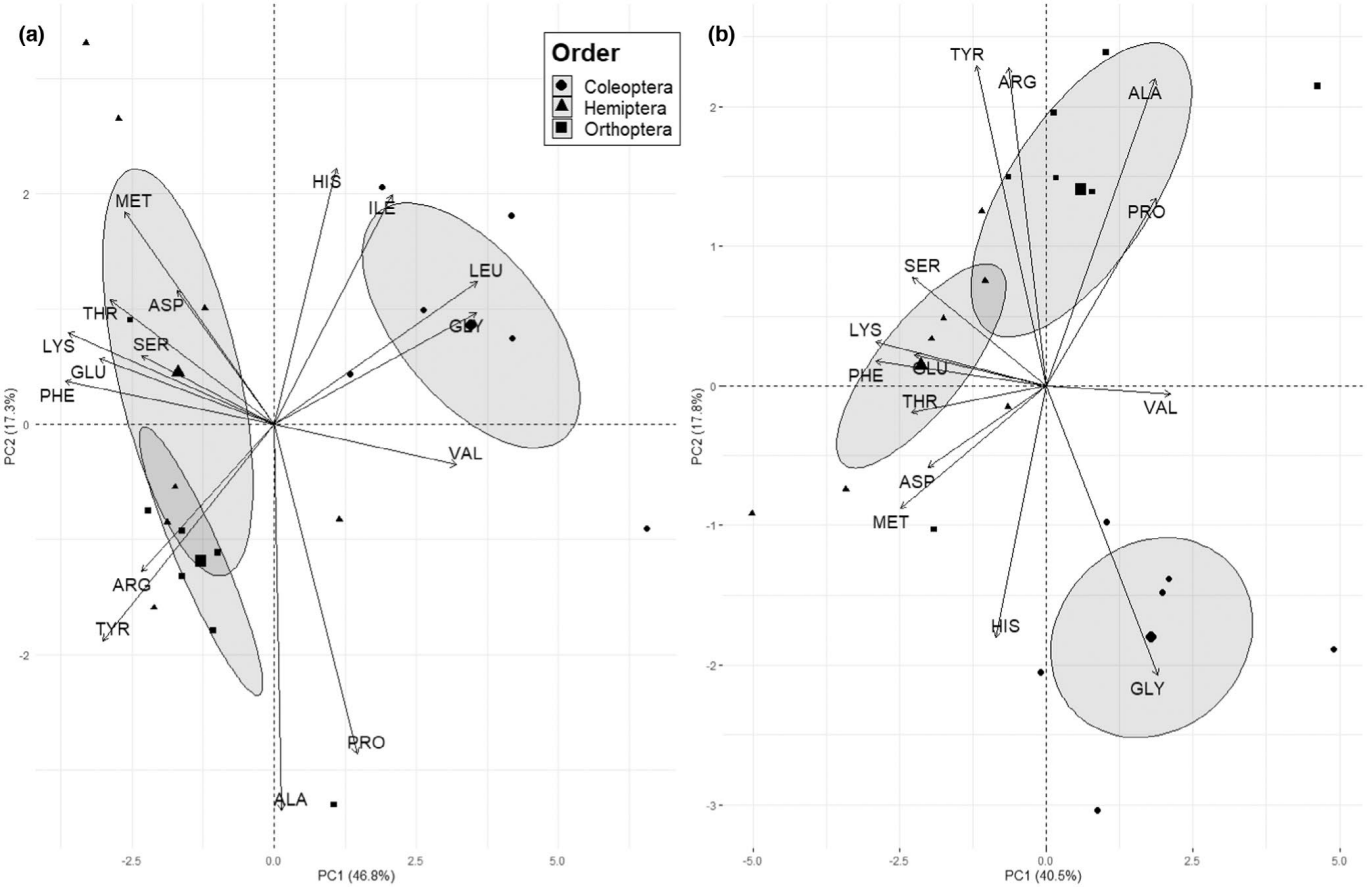
Principal component analysis of digestible amino acid balance (mg/100 mg Protein) for three arthropod orders from (a) whole arthropods and (b) digestible tissue. For (a) whole arthropods, Order significantly affected PC1 (ANOVA; *F* = 22.8, *p* = 1.6 × 10^−5^) and PC2 scores (ANOVA; *F* = 3.6, *p* = .05). For (b) digestible tissue, Order significantly affected PC1 (ANOVA; *F* = 12.25, *p* = .0005) and PC2 scores (ANOVA; *F* = 53.1, *p* = 4.9 × 10^−8^).

The PCA of whole arthropod amino acid balance (i.e., as a proportion of protein) revealed differences in overall balance between arthropod Orders (Figure 5a,b). We observed significant effects of Order for both PC1 and PC2 scores for whole amino acid balance (Figure 5a). Analysis of multivariate homogeneity of dispersions did not detect differences in dispersion between Orders (*betadisper—permutest*; permutations = 999, df = 2, *F* = 2.6, *p* = .1). PERMANOVA indicated that locations of Order centroids differed significantly in their locations in sampling space (permutations = 999, df = 2, *R* ^2^ = .6, *F* = 10.9, *p* = .001). We also observed significant effects of Order on PC1 and PC2 scores for digestible amino acid balance (Figure 5b). Analysis of multivariate homogeneity of dispersions did not detect differences in dispersion between Orders (*betadisper—permutest*; permutations = 999, df = 2, *F* = 0.1, *p* = .9). PERMANOVA indicated that locations of Order centroids differed significantly in their locations in sampling space (permutations = 999, df = 2, *R* ^2^ = 0.4, *F* = 5.1, *p* = .001).

Linear regression of PC1 scores for whole arthropods and digestible tissues revealed significant positive relationships both for content and balance with *R* ^2^ of .5 and .6, respectively (Figures S4A,B). Additionally, PC2 values of whole arthropods and digestible tissue displayed significant positive relationships as mg/100 mg dry mass and mg/100 mg protein with *R* ^2^ of .3 and .5, respectively (Figure S5A,B).

## 4 DISCUSSION

Our results provide evidence that arthropod exoskeleton and digestible tissue differ in amino acid content (i.e., mg/100 mg dry mass) and balance (i.e., mg/100 mg protein). Differences in content may be due in part to differences in the total amount of protein in each tissue, while differences in balance are due to different types of proteins that have different amino acids in each tissue. Additionally, we did not observe a relationship between digestible and exoskeleton amino acid balance across taxa. This further supports the idea that the proteins in the digestible tissue and exoskeleton are different in their amino acid balance and not related to each other within a species. These results have important implications for studying the nutritional ecology of arthropodivores.

One of the easiest ways to measure the nutritional content of an individual arthropod prey is to analyze the entire body. However, most consumers cannot digest entire arthropods, especially predators that feed using extraoral digestion in which the exoskeleton is discarded following feeding (e.g., spiders; Akaki & Duke, 1999; Barnes et al., 2019; Bell, 1990; Cohen, 1995, 1998; Foelix, 1996; Weiser et al., 1997; Walter et al., 2017). The exoskeleton content of arthropods varies widely among species from 9% to 43% of arthropod dry mass, which means that there is no average digestibility factor that can be used to calculate how much of a prey will be digested (Lease & Wolf, 2010). Also, the amino acid balance of an entire arthropod may not be a reliable predictor of the amino acid content of the digestible part of an arthropod, especially when comparing species that vary in exoskeleton or species with relatively high exoskeleton content (e.g., Coleoptera, Hymenoptera; Lease & Wolf, 2010). This complicates the study of the amino acid nutrition of arthropodivores as accurately assessing amino acid content of prey will require measuring digestible amino acid content (i.e., whole prey—exoskeleton). Fortunately, techniques for separating samples of exoskeleton from soft tissue are relatively easy and provide clean exoskeleton tissue for analysis along with a measure of the percent exoskeleton content of a sample (Cuff et al., 2021).

Interestingly, exoskeleton amino acid balance appeared more variable than that of digestible tissue (Figures 1b and 2). The digestible tissue consists of tissues and organs with similar function among arthropods (e.g., muscle, neural tissue, circulatory systems, digestive systems, etc.; Loesel et al., 2013; Peckham et al., 1992; Wirkner et al., 2013; Zhao et al., 2019). Hence, similarity among taxa in amino acid content of digestible tissue is not unexpected (see also Wybouw et al., 2016). Variation azmong taxa in exoskeleton amino acid content likely relates to different structures and functions of exoskeleton among arthropods (Zhao et al., 2019). Proteins are involved in sclerotization, or hardening, of insect exoskeleton and different proteins with different amino acid balances could contribute to relatively harder versus more flexible exoskeletons (Andersen, 1979, 2010, 2011; Sugumaran, 2022; Zhao et al., 2019).

Some amino acids may be more important for sclerotization than others. For example, tyrosine plays a key role in the initiation of sclerotization through the synthesis of acyldopamine molecules and subsequent localization of quinones (Andersen, 2010; Arakane et al., 2016), and adduction within the chitin matrix then depends on the availability and charge of amino acid side chains and isomers (Andersen, 2008, 2010; Sugumaran, 2022). Tyrosine‐derived quinones form adducts with exposed histidine, lysine, glycine, alanine isomers, and tyrosine on the surface of cuticular proteins, but the dominant composition and configuration of adducts varies across taxa (Andersen, 2007, 2008; Andersen & Roepstorff, 2007). We observed a higher proportion of histidine, tyrosine, glycine, and alanine in exoskeleton protein than in digestible tissue, but isoleucine, leucine, valine, and proline also comprised a higher proportion of exoskeleton protein. These hydrophobic (i.e., nonpolar) sidechains likely influence the internal structure of exoskeleton proteins (Zhao et al., 2019). Future studies investigating the relationships between amino acid content and the structure and function of arthropod exoskeletons would provide more insight into the nature of the variation among taxa in exoskeleton amino acid balance (e.g., Kopáček & Perner, 2016; Sterkel et al., 2016).

We also observed differences between arthropod Orders in amino acid content and balance. Thus, the observation that groups of arthropods sharing a recent common ancestor are more similar in amino acid content and balance than other groups is also supported by this work. Differences in amino acid content can be influenced by both the total percent protein of an arthropod and the amino acid balance of the proteins. Arthropod taxa are known to vary in protein content (Ramsay & Houston, 2003; Reeves et al., 2021; Simpson et al., 2015; Wilder et al., 2013). Differences in amino acid balance are also interesting because they indicate that a given mass of protein has different amino acids in different taxa. Differences in digestible amino acid content and balance are especially relevant to consumers that digest little or no exoskeleton (e.g., spiders, birds, and mammals).

In terms of digestible amino acid content (i.e., mg/100 mg dry mass), two EAAs (methionine and tyrosine) differed among Orders. Methionine is thought to be limiting for a variety of consumers (Khosravi et al., 2016; Löest et al., 2002) and may serve a variety of essential cellular, immune, or structural functions (Heiby et al., 2019; Nishimura et al., 2015; Ramsay & Houston, 2003; Roje, 2006; Zhao et al., 2012). For example, cysteine synthesis de novo requires methionine, and intermediates of this process (e.g. S‐Adenosyl methionine) are the primary source of free intracellular methyl groups for use in DNA methylation and other reactions (Niculescu & Zeisel, 2002; Roje, 2006). Methionine may also be particularly important in the diet of web‐building spiders, as methionine may mediate the energy changes associated with the secondary and tertiary structures of silk production (Heiby et al., 2019; Ramsay & Houston, 2003). While tyrosine is required for formation of acyldopamine molecules during sclerotization, degradation of excess dietary tyrosine is critical for blood‐feeding insects to avoid internal crystallization (Kopáček & Perner, 2016; Sterkel et al., 2016). The observation that fewer amino acids differed among Orders in digestible tissue compared to whole arthropods (in terms of mg/100 mg dry mass and mg/100 mg protein) again supports the idea that organs are more similar in amino acid content among species than is exoskeleton, as was observed in the first part of the study.

The finding that more closely‐related taxa are more similar in amino acid content is not unexpected. Regardless, our results confirm this observation and demonstrate that the degree of similarity and differences among taxa varies. Digestible amino acid content was similar in Coleoptera and Orthoptera, which differed from Hemiptera. However, PERMANOVA indicated that overall digestible content did not differ between Orders, which suggests that any overall differences are relatively small. Our study only examined several morphospecies within each of three Orders of insects. In addition, these diverse groups display chemical traits (e.g., defensive secretions, pheromone‐producing enzymes, venom, digestive enzyme secretions) that may differentially affect the amino acid content of body tissues (Aldrich et al., 1978; Cooper et al., 2013; Rebholz et al., 2023; Walker et al., 2017). Further work is needed to test if variation in amino acid content is related to the phylogeny of arthropods with predictable patterns of changes in amino acid content over evolutionary time. Regardless, consistent differences among arthropod taxa in amino acid content could allow for the evolution of prey choice behavior to regulate dietary amino acid intake by predators, as has been documented for some taxa (e.g., Greenstone, 1979; Murphy, 1994a, 1994b; Murphy & King, 1987; Murphy & Pearcy, 1993; Niknafs & Roura, 2018).

In conclusion, differences in the availability of amino acids among tissues and taxa of arthropods may impact consumer growth, survival, and reproduction, and merits further study. Future work describing the amino acid content and balance of arthropods would benefit from delineation between digestible tissue and exoskeleton amino acids for a broad variety of taxa. The synthetic capacity and relevant metabolic pathways for few amino acids are understood in some taxa, but knowledge connecting the amino acid needs and synthesis of arthropods to those of their consumers is largely lacking, with a few notable exceptions (Arnold et al., 2007; García‐Navas et al., 2013; Greenstone, 1979; Langlois & McWilliams, 2022). For example, tyrosine requirements for sclerotization and crystallization sensitivity may demonstrate a nutritionally important tradeoff as witnessed in blood‐feeding species (Sterkel et al., 2016). Additionally, methionine serves essential metabolic and synthetic roles, and may help mediate energy changes associated with the production of extracellular materials (e.g., spider silk; Heiby et al., 2019; Ramsay & Houston, 2003). Considering the broad diversity of consumers that rely on arthropods as food and evidence of changes in the distribution and abundance of some arthropod taxa, work investigating the availability and movement of molecules and elements between arthropod taxa and their consumers should be given high priority (Crossley et al., 2020; Fenoglio et al., 2020; Lister & Garcia, 2018; Salcido et al., 2020; Sánchez‐Bayo & Wyckhuys, 2019, 2021; Tallamy & Shriver, 2021, but see also Thomas et al., 2019; Willig et al., 2019).

## AUTHOR CONTRIBUTIONS

**Jamie T. Reeves:** Conceptualization (equal); data curation (equal); formal analysis (equal); investigation (equal); methodology (equal); project administration (equal); validation (equal); visualization (equal); writing – original draft (equal); writing – review and editing (equal). **Colton Herzog:** Conceptualization (equal); formal analysis (equal); writing – original draft (equal); writing – review and editing (equal). **Cody L. Barnes:** Conceptualization (equal); investigation (equal); methodology (equal); writing – review and editing (equal). **Craig A. Davis:** Conceptualization (equal); funding acquisition (equal); writing – review and editing (equal). **Samuel D. Fuhlendorf:** Conceptualization (equal); funding acquisition (equal); writing – review and editing (equal). **Shawn M. Wilder:** Conceptualization (equal); data curation (equal); formal analysis (equal); funding acquisition (equal); investigation (equal); methodology (equal); project administration (equal); supervision (equal); validation (equal); visualization (equal); writing – original draft (equal); writing – review and editing (equal).

### OPEN RESEARCH BADGES

This article has earned an Open Data badge for making publicly available the digitally‐shareable data necessary to reproduce the reported results. The data is available at: 10.5061/dryad.w9ghx3ft7.

## Supporting information


Appendix S1

## Data Availability

The data and code used to produce analyses in this work are available at: 10.5061/dryad.w9ghx3ft7.
