# Supplementary material for: Variation among arthropod taxa in the amino acid content of exoskeleton and digestible tissue
Source: Ecol Evol. 2023 Jul 24;13(7):e10348. doi: 10.1002/ece3.10348 (PMC10365971; doi:10.1002/ece3.10348)
Supplement: Supplementary file 1 — Appendix S1 [file ECE3-13-e10348-s001.docx]

| 1. **By Mass** | | |  | **B) By Protein** | |
| --- | --- | --- | --- | --- | --- |
| **Residue** | **F** | **p** |  | **F** | **p** |
| Ala | 0.2 | 0.6 |  | 8.5 | 0.008 |
| Arg | 33.4 | <0.0001 |  | 2.4 | 0.1 |
| Asp | 11.5 | 0.002 |  | 9.6 | 0.005 |
| Glu | 13.5 | 0.001 |  | 3.0 | 0.1 |
| Gly | 0.07 | 0.8 |  | 20.6 | 0.0001 |
| His | 0.2 | 0.7 |  | 10.7 | 0.003 |
| Ile | 0.3 | 0.6 |  | 1.7 | 0.2 |
| Leu | 0.06 | 0.8 |  | 0.2 | 0.7 |
| Lys | 18.2 | 0.0003 |  | 9.3 | 0.006 |
| Met | 21.7 | <0.0001 |  | 2.6 | 0.1 |
| Phe | 10.4 | 0.004 |  | 9.1 | 0.006 |
| Pro | 0.1 | 0.7 |  | 5.6 | 0.03 |
| Ser | 4.6 | 0.04 |  | 0.07 | 0.8 |
| Thr | 8.5 | 0.007 |  | 6.8 | 0.02 |
| Tyr | 6.4 | 0.02 |  | 5.1 | 0.03 |
| Val | 0.5 | 0.5 |  | 6.4 | 0.02 |

**Table S1.** Test statistics for Levene’s test performed on individual amino acids to test differences in variance between digestible tissue and exoskeleton. Tested df = 1 and residual df = 24 for all comparisons. Amino acid data were calculated as **A)** a proportion of dry mass (mg /100 mg dry mass) and **B)** a proportion of total protein (mg /100 mg protein). P-values < 0.05 are considered significant.

| 1. **Whole Body** | | |  | **B) Digestible** | |
| --- | --- | --- | --- | --- | --- |
| **Residue** | **F** | **p** |  | **F** | **p** |
| Ala | 1.8 | 0.2 |  | 0.9 | 0.4 |
| Arg | 0.5 | 0.6 |  | 0.3 | 0.7 |
| Asp | 1.7 | 0.2 |  | 1.3 | 0.3 |
| Glu | 0.8 | 0.5 |  | 0.4 | 0.7 |
| Gly | 3.6 | 0.05 |  | 0.2 | 0.9 |
| His | 0.8 | 0.5 |  | 0.7 | 0.5 |
| Ile | 1.6 | 0.2 |  | --- | --- |
| Leu | 2.8 | 0.1 |  | --- | --- |
| Lys | 1.1 | 0.4 |  | 0.7 | 0.5 |
| Met | 0.9 | 0.4 |  | 0.7 | 0.5 |
| Phe | 1.4 | 0.3 |  | 1.4 | 0.3 |
| Pro | 0.8 | 0.4 |  | 1.1 | 0.3 |
| Ser | 0.7 | 0.5 |  | 0.7 | 0.5 |
| Thr | 2.4 | 0.1 |  | 0.5 | 0.6 |
| Tyr | 4.1 | 0.04 |  | 4.0 | 0.04 |
| Val | 0.07 | 0.9 |  | 0.1 | 0.9 |

**Table S2.** Test statistics for Levene’s test performed on individual amino acids to test differences in variance between three orders of arthropod. Tested df = 2 and residual df = 17 for all comparisons. Amino acid data were calculated as a proportion of dry mass (mg /100 mg Dry Mass) from **A)** whole arthropods and **B)** metabolizable tissue. P-values < 0.05 are considered significant.

| 1. **Whole Body** | | |  | **B) Digestible** | |
| --- | --- | --- | --- | --- | --- |
| **Residue** | **F** | **p** |  | **F** | **p** |
| Ala | 0.4 | 0.7 |  | 0.5 | 0.6 |
| Arg | 0.1 | 0.9 |  | 0.1 | 0.9 |
| Asp | 1.9 | 0.2 |  | 1.3 | 0.3 |
| Glu | 0.1 | 0.9 |  | 0.5 | 0.6 |
| Gly | 23.9 | <0.0001 |  | 1.1 | 0.3 |
| His | 12.7 | 0.0004 |  | 19.1 | <0.0001 |
| Ile | 21.3 | <0.0001 |  | --- | --- |
| Leu | 2.5 | 0.1 |  | --- | --- |
| Lys | 0.8 | 0.5 |  | 0.1 | 0.9 |
| Met | 0.8 | 0.5 |  | 1.3 | 0.3 |
| Phe | 0.7 | 0.5 |  | 0.2 | 0.9 |
| Pro | 2.3 | 0.1 |  | 0.5 | 0.6 |
| Ser | 1.4 | 0.3 |  | 0.2 | 0.8 |
| Thr | 1.2 | 0.3 |  | 0.4 | 0.7 |
| Tyr | 4.0 | 0.04 |  | 3.1 | 0.1 |
| Val | 2.3 | 0.1 |  | 3.2 | 0.1 |

**Table S3.** Test statistics for Levene’s test performed on individual amino acids to test differences in variance between three orders of arthropod. Tested df = 2 and residual df = 17 for all comparisons. Amino acid data were calculated as a proportion of total protein (mg /100 mg protein) from **A)** whole arthropods and **B)** metabolizable tissue. P-values < 0.05 are considered significant.

**
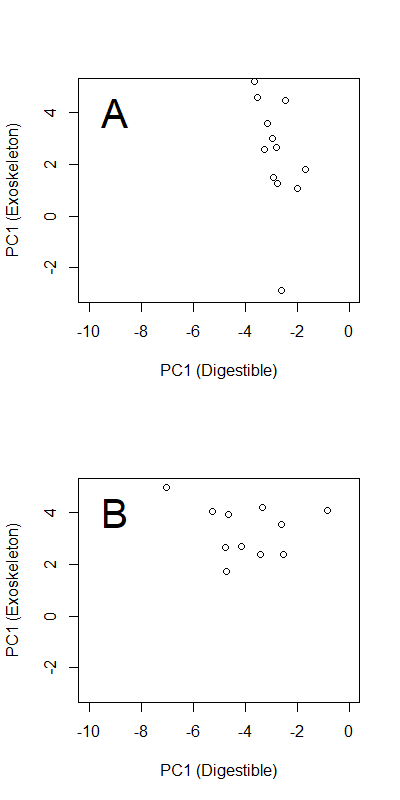
**

**Figure S1.** Scatterplot of principal component 1 (PC1) values from tissue dataset PCAs. Plots are displayed as **A)** balance (mg /100 mg Protein) and **B)** content (mg /100mg Dry Mass) in PC space. Linear regressions did not indicate a significant relationship between tissue type for content (A; p = 0.8) or balance (B; p = 0.4).


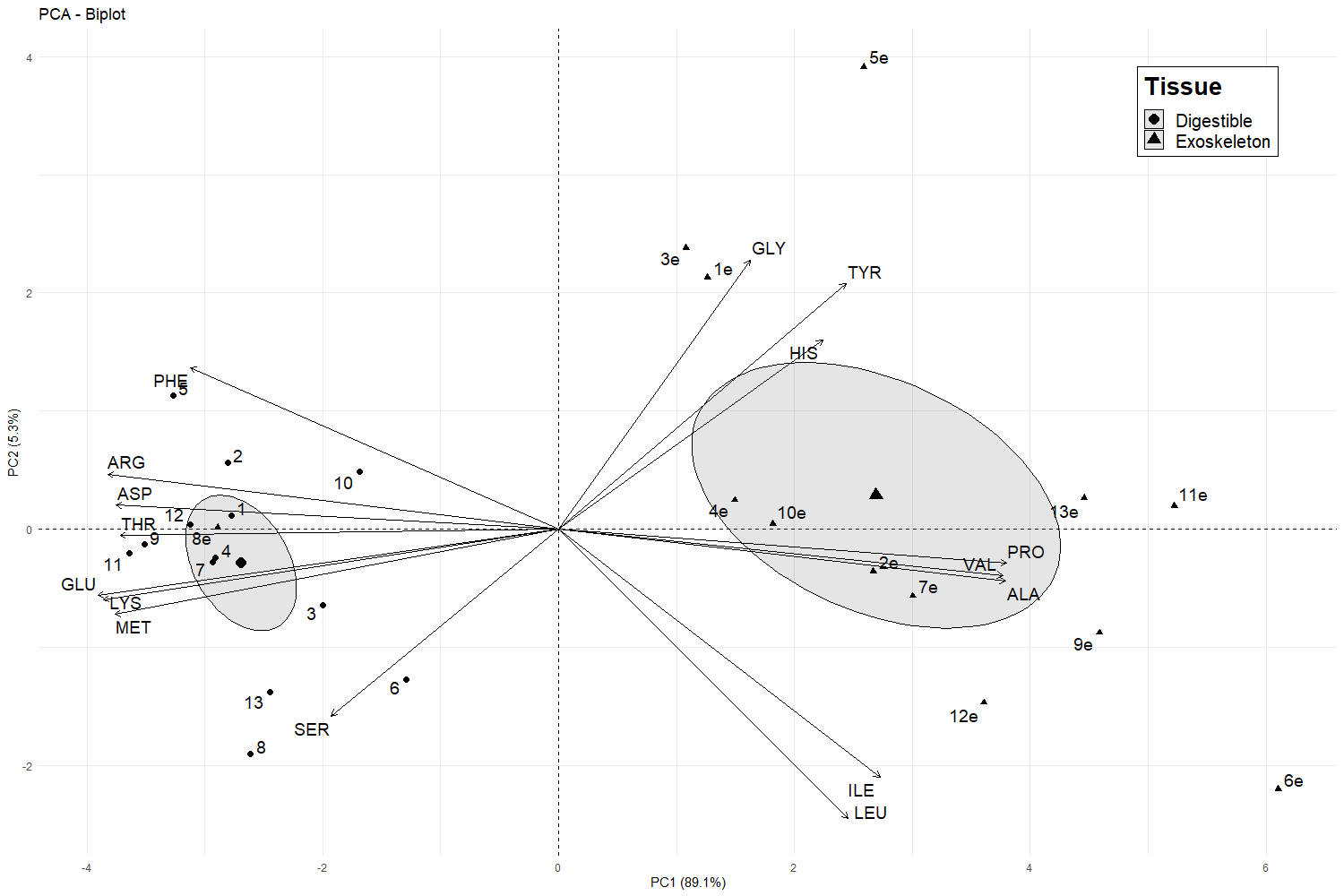


**Figure S2.** Principal component analysis of metabolizable and exoskeleton amino acid balance of 16 individual amino acids as a proportion of dry mass (mg /100 mg Dry Mass). Large points indicate group centroids, and ellipses indicate 95% confidence intervals. Point label indicates corresponding arthropod Order: 1) Araneae, 2) Blattodea, 3) Coleoptera, 4) Diptera, 5) Ephemeroptera, 6) Hemiptera, 7) Hymenoptera, 8) Isopoda, 9) Lepidoptera, 10) Mantodea, 11) Odonata, 12) Orthoptera, and 13) Phasmatodea. Inclusion of “e” in point label indicates exoskeleton. A significant effect of tissue was observed on PC1 (ANOVA; F = 34.0, p < 0.0001).


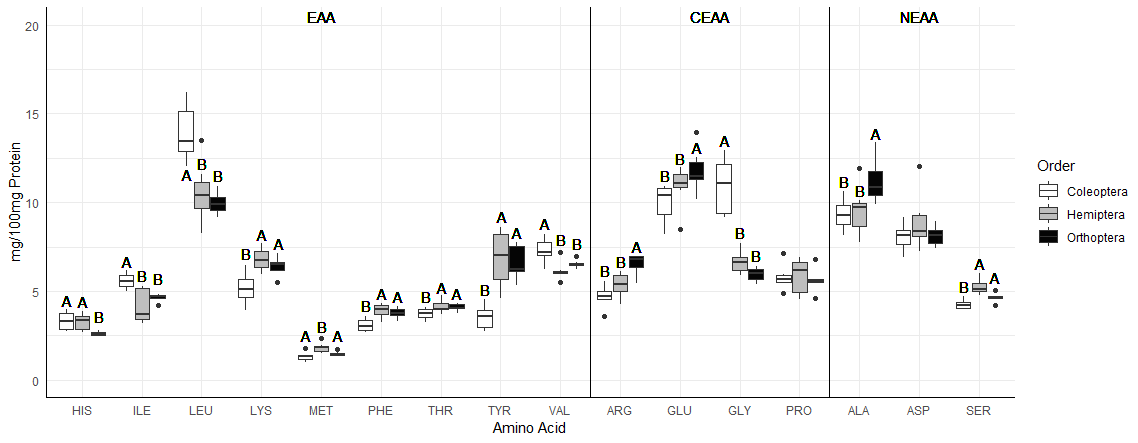


**A)**

**B)**


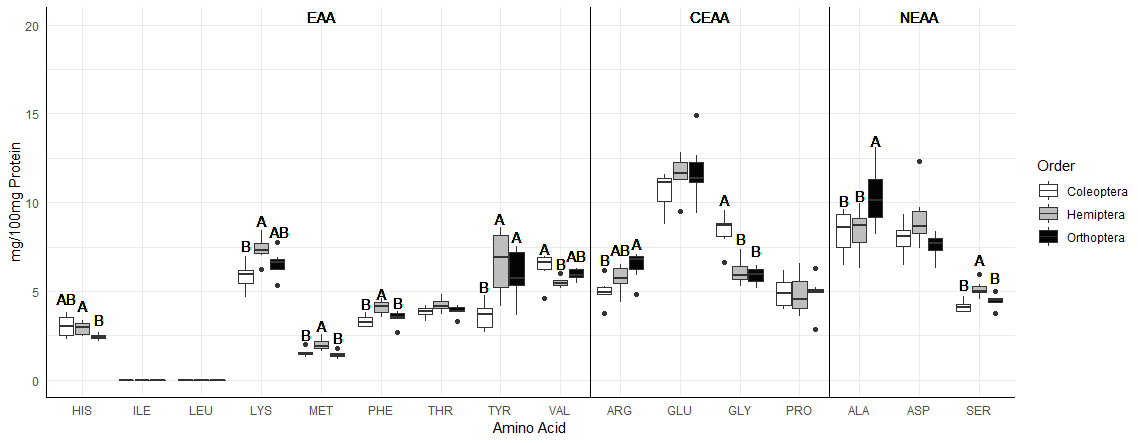


**Figure S3.** Balance of individual amino acids measured in three Orders of arthropod: Coleoptera (*n* = 6), Hemiptera (*n* = 7), and Orthoptera (*n* = 7). Amino acid data were calculated as a proportion of protein (mg /100 mg Protein) from **A)** whole arthropods and **B)** digestible tissue. Groups within an individual amino acid not connected by the same letter differ significantly, but letters are not comparable across amino acids. Unlabeled amino acids were not affected by Order.


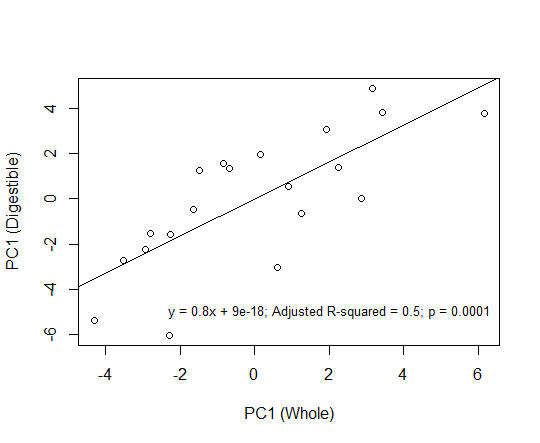

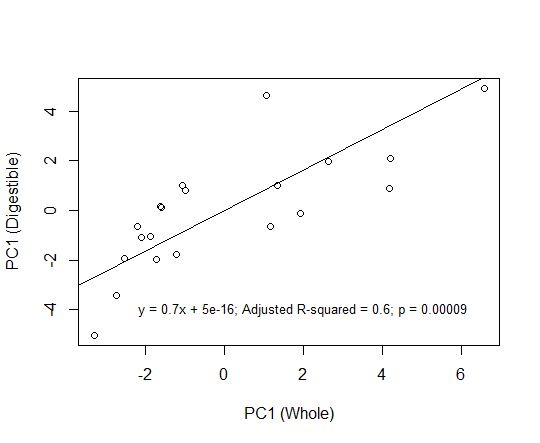


**B)**

**A)**

**Figure S4.** Linear regression of PC1 computed from whole arthropod and digestible tissue PCAs as **A)** mg /100mg Dry Mass and **B)** mg /100mg Protein.


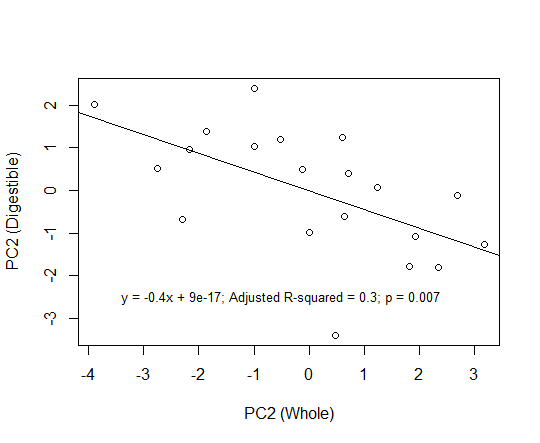

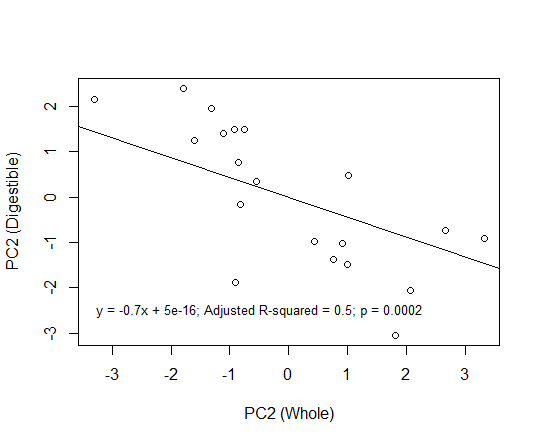


**A)**

**B)**

**Figure S5.** Linear regression of PC2 computed from whole arthropod and digestible tissue PCAs as **A)** mg /100mg Dry Mass and **B)** mg /100mg Protein.
